# Hemicrania continua- *building on experience and clinical science*

**DOI:** 10.1186/1129-2377-15-9

**Published:** 2014-02-13

**Authors:** Peter J Goasdby

**Affiliations:** 1 Headache Group, Department of Neurology, University of California, San Francisco, USA; 2 NIHR Wellcome Trust Clinical Research Facility, King's College, London, UK

## Abstract

This is a reply letter to Letter to the ‘Editor Hemicrania continua: towards a new classification?’ Fabio Antonaci and Ottar Sjaastad The Journal of Headache and Pain Citation: 2014, 15:8.

## 

To the Editor

The third edition of the International Classification of Headache Disorders- ICHD-III has recently been published in beta [1], or test, format, to focus developments in headache nosology. The beta version is to “test” and for the “correction of mistakes”, so your writers’ concerns [2] need to viewed in that context.

The authors commence with their underlying rationale: “Naturally, later reported cases, carrying this appellation, should conform with the original description of this clinical constellation of symptoms and signs”, referring to the cases of Sjaastad and Spierings [3]. Now the ICHD-III beta committee could pause there and say the new definition conforms by the definition of “to make like” (Oxford English Dictionary), since like gives some flexibility, although that may be seen a rather insouciant, if not specious, approach. The extent to which the proposed definition renders cases *like* the original is at issue. Since I have not seen the original cases, and I doubt the writers have seen any of those I have seen, perhaps this will not be easily resolved.

*Are the movement criteria- 3.4 C2 wrong and lead to mis-diagnosis?* This writers’ view is that 3.4 C2 should not be sufficient in the absence of any cranial autonomic symptoms. We have seen and documented cases with strictly lateralized continuous headache, a placebo-controlled response and then oral response to indomethacin, who did not have cranial autonomic symptoms, but fulfilled C2 [4]. The writers mention cluster headache in this context as if patients fulfilling the new criteria could have that condition. However, patients falling into the new classification would be treated correctly, as our index cases were, and not with oxygen, sumatriptan, verapamil or lithium, by a mis-diagnosis the writers have opined on recently; a text that makes good reading [5].

*How important is the indomethacin effect?* The new criteria insist on an indomethacin effect. It has always been troublesome, however, until we understand the biology, it seems clinically and nosologically sensible; it is clearly stated in the new criteria. The acronym NIRCH cannot apply to the cases classified with the new criterion, “Non” is not an option. The writers state the indomethacin dose can vary; that is certainly this correspondent’s experience, and is covered explicitly by the statement- *Smaller maintenance doses are often employed* (3.4 Note 1). To say 100–200 mg of indomethacin tests the general analgesic properties is inconsistent with the fact that many patients do not respond at all [6]; the inclusion of placebo in the test we have developed gives these data a strength absent in previous studies.

*Migrainous features and side-locking: what is the issue?* The writers mention migrainous features and vascular components, neither are mentioned in the new criteria; the latter is unreferenced, thus hard to understand in this context. Regarding side-locking, again we have documented cases otherwise typical with a clear placebo-controlled indomethacin effect [4] that are not side locked. Indeed, this correspondent has also seen strictly lateralized headache that swaps sides and is not indomethacin responsive: which is the greater feature with which to classify such cases?

*Are cranial autonomic features part of hemicrania continua?* The writers describe the inclusion of certain cranial autonomic features as a “blunder”, specifically they write: “Forehead/facial sweating is not part of the HC-picture.” I would invite them to consider the original description [3]: “His right eye would then become red and sensitive to light. It would also tear, and there would be profuse right-sided forehead perspiration.” One would predict the cranial autonomic activation to vary and there is a good physiological basis for this [7].

*How should we define the length of a remission?* The writers feel the Committee has been too lenient with a remission of one day. I would not quibble; one would invite studies of the length of remission of cases with clear-cut indomethacin effects to see what is a reasonable interval. Reductionist logic tells you any length spontaneous remission deserves our attention in order to learn from it.

I would submit the diagnosis has not changed, rather our understanding is evolving. A rather wise and astute clinician once wrote: “One can, however, hardly expect all the facets of this headache to be established by now. Only experience will clarify the entire panorama of symptoms and signs in this headache; only then will its nosologic position be ascertained” [8]. The ICHD Section III sub-committee would entirely agree and continues to attempt this process of clarification so ably commenced by your senior writer.

## Competing interests

The author declares that he has no competing interests.
