# Correction: Hemicrania continua- *building on experience and clinical science*

**DOI:** 10.1186/1129-2377-15-29

**Published:** 2014-05-15

**Authors:** Peter J Goadsby

**Affiliations:** 1 Headache Group, Department of Neurology, University of California, San Francisco, USA; 2 NIHR Wellcome Trust Clinical Research Facility, King's College, London, UK

## Correction

The original version of this article [1] unfortunately listed the author’s name as ‘Peter J Goasdby’. The correct author name is Peter J Goadsby. The publisher would like to apologise to the author for this error and the inconvenience caused.
